# Giving voice to the voiceless: Understanding the perceived needs of dementia family carers in Soweto, a South African township

**DOI:** 10.1177/14713012241234155

**Published:** 2024-02-14

**Authors:** Aqeela Mahomed, Chrisma Pretorius

**Affiliations:** Department of Psychology, 26697 Stellenbosch University, South Africa

**Keywords:** unmet needs, dementia, family carers, psychoeducation, community mobilization, national dementia plan

## Abstract

This qualitative study aimed to provide family caregivers with an independent platform to reflect on and identify their needs in the role of dementia caregiver. Thirty caregivers were interviewed using a semi-structured approach, and data analysis followed a reflective thematic analysis method. The study revealed that Black African caregivers in townships require sufficient information and orientation to dementia-specific services, psychoeducation on dementia as a disease and its behavioural manifestations, as well as practical skills to manage the disease process. Caregivers expressed the need for in-depth, accessible education to boost their confidence and resilience in handling the challenges of dementia caregiving. They also proposed community initiatives to raise awareness, promote knowledge, and facilitate early detection and diagnosis of dementia. Additional needs included informational and educational workshops, resources like transportation services and helplines, day care facilities, media campaigns, and collaboration with the government for funding and policy change. New caregivers were advised to seek comprehensive education, support, and services while preserving the dignity of their family members with dementia. Remarkably, the identified needs and community initiatives aligned with the priority areas outlined by ADI for a National Dementia Plan, which South Africa currently lacks. The study highlights the importance of developing a National Dementia Plan in South Africa through collaboration among stakeholders, including communities, policy-makers, and multidisciplinary healthcare teams, while ensuring that individuals and families affected by dementia have a voice.

## Introduction

According to the World Health Organization (WHO, 2018), there are 50 million individuals living with dementia globally, with prevalence rates expected to rise in low and middle-income countries (LMICs). Among these countries is South Africa, where De Jager et al. (2017) reported an estimate of 352,000 older people over the age of 60 who are currently living with dementia. This is almost double the South African figure reported by Alzheimer’s Disease International (ADI, 2016). Of concern, the country’s healthcare system is ill-equipped to meet the demands that this disease imposes (Kalula & Petros, 2011). Challenges include the shortage of medical professionals qualified to work with geriatric populations, a lack of education and training of health professionals to identify and treat the disease and insufficient resources to manage patients diagnosed with dementia (De Jager et al., 2015; Kalula, 2013; Kalula & Petros, 2011; Mahomed & Pretorius, 2020). As a result, people living with dementia and their families who present to primary healthcare facilities are directed back to their home environment to receive familial care and support (Kalula et al., 2010; Koyanagi et al., 2019; Mwendwa et al., 2022; Patel & Prince, 2001; Prince et al., 2007; Spittel et al., 2021). Thus, family caregivers are expected to take care of their family members’ basic and treatment needs such as activities of daily living, healthcare, financial and travel requirements (Jennings et al., 2015; McCabe et al., 2016; Mwendwa et al., 2022; Stein et al., 2020; White et al., 2018) – without any remuneration in this regard (Bosch, 2014; Kehoua et al., 2019).

Over time, these stressors have been well-documented in the literature as a precursor to caregiver burden as the demands of caregiving increase (Black et al., 2013; Winblad et al., 2016), together with adverse effects on caregiver’s psychological, emotional, physical health (Ransmayr et al., 2018; Roberts & Struckmeyer, 2018). Furthermore, there is evidence to suggest that factors such as unmet needs, lower socioeconomic status or income, geographic or service delivery issues and resource constraints (Gaugler et al., 2004; Jennings et al., 2015; Pinquart & Sorensen, 2007; Robison et al., 2009; Su & Chang, 2020; Vitaliano et al., 2005; Wei et al., 2022) exacerbate caregiver burden (Cauli et al., 2021; Oliveira et al., 2020; Pasquini et al., 2022). Focussing on unmet needs in particular, research suggests that caregiver needs are not met by healthcare professionals and a disparity exists between services and support given to caregivers versus the services that they identify as needed (Kokorelias et al., 2021; McCabe et al., 2016; Novais et al., 2017; Phillipson et al., 2014; Valer et al., 2015).

Dementia caregiving literature, both globally and in South Africa, has primarily examined the stressors that contribute to caregiver distress, service needs in light of resource constraints, the lack of services to support caregivers and the need to intervene with both caregivers and diagnosed family members to facilitate improved overall functioning and service utilization (Aledeh & Habib Adam, 2020; Bosch, 2014; Burns, 2000; Gurayah, 2015; Mahomed & Pretorius, 2020; Manderson et al., 2022; Mittlman et al., 2004; Ransmayr et al., 2018; Springate & Tremont, 2014; Van der Poel & Pretorius, 2009). Of note, there have been studies that highlight the notion that the unmet needs of caregivers could be attributed to the discrepancies between professionals’ assessments of caregiver needs and the perceived needs of caregivers themselves (Cassidy, 2019; Cohen-Mansfield & Frank, 2008; Coley et al., 2022; Haas et al., 2021; Orrell et al., 2008; Zwingmann et al., 2020). Additionally, a systematic review by McCabe et al. (2016) found that “the majority of studies that have examined the needs of family caregivers of people with dementia have been quantitative studies” (p.71). To address this gap, whilst considering the additional difficulties that caregivers in a township may experience such as poverty and destitution, resource constraints, lack of support and services and a myriad of difficult social conditions (Chen et al., 2018; García-Mochón et al., 2019; Kalula & Petros, 2011; Visser & Moleko, 2012), this study explored the subjective needs of dementia family caregivers in Soweto, a township in South Africa – rendering this study the first of this nature in the country.

## Methods

### Sample and recruitment

This study forms part of a larger study that explored the lived experiences of dementia family caregivers in a Black African township (Mahomed & Pretorius, 2022). Ethical clearance was granted for this study by the Research Ethics Committee at Stellenbosch University (PSY-2019–10582). Researchers of this study collaborated with *Alzheimer’s South Africa,* (henceforth referred to as *Alzheimer’s SA*) - a Non-Governmental Organization (NGO) actively involved in the psychoeducation of caregivers with the aim of facilitating a better understanding of the disease as well as ensuring the provision of improved care responses. This association also provides support groups for caregivers as a supportive resource (Kalula & Petros, 2011). Purposive sampling techniques were used to identify thirty family caregivers who met the inclusion criteria for the study as outlined in Table 1. These criteria was shared with the *Alzheimer’s SA* coordinator, who established initial contact with suitable participants to introduce the research objectives and explain the interview process. This was meant to allay any fears or hesitance associated with the study.

**Table 1.** Inclusion Criteria for the study.

1. Caregivers should have already received a diagnosis of dementia for their family member
2. Caregivers should be a family member of the person with dementia
3. In addition to the above, caregivers should reside in the same home in Soweto as their family member diagnosed with dementia
4. Participants should be primarily responsible for activities of daily living for their family member such as bathing, feeding, dressing, toileting and medication management
5. Family caregivers should be taking care of their family member for at least one year
6. Caregivers should be comfortable conversing in English
7. Caregivers should be 18 years or older

Caregivers who were interested to participate in the research were contacted telephonically by the first author to invite them to participate in the research. During this process, caregivers were informed that their choice to participate in the research was voluntary and to clarify that there will be no costs to them. Participants were reimbursed for the travel costs that were incurred for transportation to the *Alzheimer’s SA* office.

After receiving verbal consent, face-to-face interviews were scheduled at the *Alzheimer’s SA* office to suit caregivers’ schedules. Written consent was obtained before data collection commenced. To ensure confidentiality and the protection of the participants’ identities, code names, for example ‘Family Caregiver 1’ (FC1), was used instead of using any real identifying data and any identifying information was omitted. Participant characteristics are outlined in Table 2.

**Table 2.** Sample characteristics.

FC	Gender	Age	Dementia Individual’s relationship to caregiver	Caregiving dyad	Duration of caregiving (years)	HLOE
1	Female	30	Paternal Grandmother	Granddaughter caring for Grandmother	3	Tertiary
2	Female	50	Mother	Daughter caring for Mother	1	Tertiary
3	Male	28	Mother	Son caring for Mother	1	Matric
4	Male	58	Wife	Husband caring for Wife	5	Grade 7
5	Female	23	Mother	Daughter caring for Mother	2	Tertiary
6	Female	54	Mother	Daughter caring for Mother	5	Grade 11
7	Female	39	Mother	Daughter caring for Mother	6	Tertiary
8	Female	41	Mother	Daughter caring for Mother	3	Tertiary
9	Female	47	Mother	Daughter caring for Mother	2	Tertiary
10	Female	58	Mother	Daughter caring for Mother	2	Grade 10
11	Female	66	Husband	Wife caring for husband	3	Tertiary
12	Male	47	Mother	Son caring for Mother	2	Matric
13	Female	20	Grandmother	Granddaughter caring for Grandmother	2	Matric
14	Male	22	Grandmother	Grandson caring for Grandmother	2	Matric
15	Female	51	Mother	Daughter caring for Mother	3	Tertiary
16	Male	30	Mother	Son caring for Mother	6	Matric
17	Female	52	Mother	Daughter caring for Mother	5	Matric
18	Male	51	Mother	Son caring for Mother	5	Grade 11
19	Female	74	Husband	Wife caring for husband	4	Tertiary
20	Female	40	Mother	Daughter caring for Mother	1	Grade 10
21	Female	49	Mother	Daughter caring for Mother	2	Grade 11
22	Male	74	Wife	Husband caring for wife	2	Tertiary
23	Female	54	Mother	Daughter caring for Mother	4	Tertiary
24	Female	60	Sister	Sister caring for Sibling	3	Tertiary
25	Female	72	Husband	Wife caring for husband	1.5	Grade 9
26	Female	50	Mother	Daughter caring for Mother	5	Matric
27	Male	54	Father	Son caring for Father	4	Grade 11
28	Female	63	Husband	Wife caring for husband	1	Grade 10
29	Male	40	Mother	Son caring for Mother	1	Matric
30	Female	64	Husband	Wife caring for husband	3	Tertiary

*Note.* FC = Family Caregiver; HLOE = Highest level of education; Grade = level at school; Matric = refers to graduating from high school in South Africa; Tertiary = refers to college education.

### Data Collection

In-person, individual interviews were conducted following the semi-structured interview guide shown in Table 3. Where appropriate, prompting was utilized to elicit a complete understanding of caregiver needs. Each interview spanned between 60 and 90 minutes. Participants gave their permission to audio record their interviews so that they may be transcribed verbatim. In order to assure the accuracy and impartiality of participants’ narratives, interviews were transcribed by a transcription service due to time constraints.

**Table 3.** Semi-structured interview guide.

1. Since you started taking care of your family member with dementia, have you had enough support?
2. What services were available to you when your family member first got diagnosed?
3. How did you find out about these services?
4. Have you used any of the services that are available?
5. If so, which services have been the most helpful and why?
6. What do you think the community needs to help those who are caregivers of patients with dementia?
7. What do you need as a caregiver, to make this task more manageable?
8. What would you recommend to new caregivers of patients with dementia?
9. What would you say is the most important thing for them to know?

## Data analysis

Reflexive Thematic Analysis (RTA), as described by Braun and Clarke (2006; 2019), was used to analyze the data. This process involved creating early codes after familiarizing oneself with the data, developing themes, considering possible topics, identifying and defining themes and completing the report. Table 4 gives a brief description of each procedure.

**Table 4.** Data analysis process.

Stage	Analysis process
Familiarisation with the data	To confirm the accuracy of the transcriptions, the authors immersed themselves in the data by reading the transcripts and concurrently listening to the audio. This allowed the authors to recollect the interview’s ambience and make note of any reflections or observations that surfaced; with each fresh reading and audio listen, new insights were gained
Generating initial codes	The transcripts and audio recordings were mirrored accurately in the detailed notes. According on the information the participant gave, this generated initial codes to organize the data into relevant clusters. The qualitative data analysis program ATLAS.ti (2021) was utilized to facilitate this stage and aid in the subsequent theme process due to the massive volume of data gathered
Generating initial themes	The links between the detected codes were grouped into themes using ATLAS.ti (2021) based on common conceptualizations. The meanings of the codes and their connections between them were decrypted, and each group was given a descriptive name
Reviewing potential themes	The full data set was examined, and patterns in the coded data were found. Themes that overlapped were combined and improved
Defining and naming themes	In answer to the research questions, a list of key themes and subthemes was categorised, and the narrative of the themes was recognized and conceptualized within the larger tale of the data set.
Producing the report	We summarized our key findings in answer to our study questions using brief transcript excerpts to give a narrative overview of participant data

### Trustworthiness

This study employed three strategies to enhance the credibility of its findings while maintaining rigor in data interpretation (Long & Johnson, 2000). First, the researcher engaged in reflective practices, considering not only the perspectives of the participants but also their own thoughts and biases. To facilitate this, a personal journal was maintained throughout the interview process, allowing for self-reflection. Regular discussions among co-authors ensured that any subjective biases were identified and minimized. Second, response validation was employed to verify the accuracy and clarity of the collected information. Participants were given the opportunity to confirm the meanings they conveyed after each interview, ensuring that their intended messages were accurately represented. Lastly, peer debriefing was conducted to incorporate additional insights and alternative perspectives. This involved ongoing discussions among the authors, verifying the data, themes, and interpretations throughout the entire interview process (Long & Johnson, 2000). By employing these three methods, the study aimed to uphold the credibility of the findings and maintain a robust research process.

## Findings

The following four major themes emerged from the data analysis: (1) Assessing Community Resources, (2) Identifying Caregiver Needs, (3) Mobilizing the Community, and (4) From Caregiver to Caregiver. To illustrate these concepts, a few quotes with participant characteristics are presented. As supplemental information, a thorough list of quotes that serve as illustrations have been made available.

### Theme 1: Assessing community resources

This theme depicts the level of community resources that caregivers had access to and utilized at the time of this study via the subthemes hereunder namely, *Accessing services to obtain a diagnosis*, *Services after diagnosis*, *Helpful Services* and *No service use*.

#### Accessing services to obtain a diagnosis

Almost all caregivers described accessing public healthcare facilities as their first point of contact when they noticed that their family member was “not okay.” Specifically, sixteen caregivers took their family members to their local day clinic and twelve caregivers accessed a hospital in Soweto, where a probable diagnosis was obtained. Of note, this was not a seamless process, as caregivers described vacillating between clinics and the hospital before they received appropriate assistance (Q1-Q3). Only two caregivers consulted with private health professionals, who then referred them to support services such as *Alzheimer’s SA* (Q4).

“Okay. That’s when we say “okay, she’s not okay” and then we went to the clinic but still it took time because you know clinics, they didn’t like transfer us to the doctor that she is in right now. It took us surely a year doing the same thing over and over again” (Q2 - 54 year old, female, unemployed)

“Because first, we didn’t know. First, I remember I used to take her to [the hospital], and then from [the hospital], they didn’t help us, and then back to the clinic. And then we went to the clinic, went to the clinic, until one doctor referred us to the mental [health] clinic” (Q3 - 58 year old, female, employed)

“Like we went to a doctor, there’s a doctor she went to who recommended a psychologist, and when we got that, we then heard of this a week later…. so we haven’t really had any services – this is our first sort of contact…” (Q4 - 30 year old, female, employed)

#### Services after diagnosis

Most caregivers had no knowledge of any dementia-related services after they received a diagnosis at either their clinic or hospital and did not receive any guidance thereof (Q5). A caregiver who received medication from the clinic stated that it had “no effect” (Q6) on their family member’s condition. Other caregivers expressed the sentiment, “There isn’t much information out there – there is nowhere to go” (Q7). Of note, eleven caregivers explained that they too, had no knowledge of services upon diagnosis. However, they discovered *Alzheimer’s SA* either through their own internet search, were referred by others or watched a television programme, where they recognized similar symptoms and contacted the number provided at the end of the episode (Q8-Q10).

“No there were no services, the only thing is they brought her some tablets she drank them but they had no effect you see” (Q6 - 28 year old, male, unemployed)

“None. Nothing. Nothing at all. Everything I found I had to look for, I had to really look for it (Q9 - 40 year old, female, unemployed)

“Where I saw a program on TV and I think that’s everything there it was almost similar to what I was experiencing at home, and I got the name there of the people that were responsible and I called them” (Q10 - 60 year old, female, unemployed)

#### Helpful Services

When caregivers were asked if they had experienced any services that have helped them, caregivers identified the services provided by *Alzheimer’s SA*. Many caregivers described the psychoeducation given either at a clinic intervention or through caregiver workshops, where they were given didactic, practical and management tools specific to taking care of someone with dementia (Q11-Q12). Furthermore, some caregivers experienced the home visits and support groups by *Alzheimer’s SA* as valuable as caregiver families were psycho-educated at home (Q13-Q14) and caregivers felt supported and able to better handle difficult emotions by sharing with other caregivers in the group (Q15-Q16).

In addition, although one caregiver did not find medication effective as described above, a few other caregivers did receive medication that made their family members’ behaviour “more manageable”. Therefore, these caregivers identified this as a “helpful” service by their local clinic (Q17-Q18).

“It was teaching us about the dementia, how to take care of the person who has dementia and how the difference between a normal, what do you call, old person and a dementia person. Yes. in the normal for, if you forget, it, sometimes its normal and then there’s a difference between a person who forgets with dementia. That person will forget and not knowing it all away where is something. It is teaching about how to take care of elderly people. How to wash them and how to give them food, water, maybe sometimes you should use colouring things. So that the person can enjoy the water. It’s so, it was so good” (Q11 - 54 year old, male, unemployed)

“Non-medical services, I think the counselling, being able to talk to someone who understands like what’s going on. I like that they come to you to the patient house. They talk to the family. I actually invited her sister and her husband to come and also listen try to a better understanding of what, what it is and how to communicate with that person from now, just to make things better. And the support groups help a lot also” (Q13 - 50 year old, female, employed)

“Yeah, before yeah I don’t want to lie. Before the clinic it was helpful with their pills, because she was missing, going up and down, they give me pills” (Q17 -51 year old, female, unemployed)

#### No service use

Very few caregivers in this study stated that they do not use any of the services at *Alzheimer’s SA.* With the exception of two caregivers – who were unaware of the support groups (Q19) – it appeared that caregivers did not need this service (Q20-Q21) or find it useful because “it’s the same people and it’s the same stories” (Q22).

“I did not know about the support groups” (Q19 - 40 year old, male, unemployed)

“Yes, she told us that there is support groups and whatever but we have never been in one of them” (Q20 - 60 year old, female, unemployed)

“No, I didn’t ask for help though” (Q21- 47 year old, female, employed)

“Well, I find that I don’t need to because it’s the same people and it’s the same stories” (Q22 - 49 year old, female, employed)

### Theme 2: Identifying caregiver needs

#### Respite care

Half the caregiver group indicated that they needed assistance to allow for a “break” in their daily caregiving tasks. They expressed needing help in their homes to “rejuvenate” because “sometimes it’s exhausting to take care” (Q23) of their family members. Furthermore, caregivers also expressed the wish to socialize and the need for some space to themselves to go where they choose – “somebody to help” at home will enable these needs (Q24-Q25).

“…sometimes I need to have my own space. You know so that I can rejuvenate myself, you know because sometimes it is exhausting to take care of that person and even weekends you are still there, we have to go to church together and come back. Monday, Tuesday, Wednesday, Thursday. You know you are doing the same thing every day. So you don’t get rest…” (Q23 – 47 year old, female, unemployed)

#### Psychoeducation and skills training

The majority of caregivers identified the need to be taught more about dementia as a disease, its manifestations and how it might affect their family member’s behaviour (Q26-Q27). Furthermore, they expressed these needs to equip them with the necessary information and practical skills to understand *how* to “treat” (Q28), and respond to their family members’ behaviours, as well as to facilitate better awareness to identify and attune to what their needs as caregivers are (Q29). In addition, caregivers need to be able to have “a lot” of information on hand for quick reference on how to take care of [a] situation when it happens” (Q30) and “resources that make [their] lives easier” (Q31).

“I think it’s education, education for the caregivers, because when you ask me what do I need to help take care of mum, maybe with the right education or enough knowledge of the illness and what is being used with other patients at home, then I would know that, okay, we don’t have this, we need this, we don’t have this, we need that” (Q29 - 23 year old, female, unemployed)

“A lot of information, and if I have questions, being able to access help quick, resources that will make our lives easier” (Q31 - 47 year old, female, unemployed)

#### Emotional support

Some caregivers communicated that they needed counselling (Q32) to feel emotionally supported as they struggle to cope with the demands of caring for their family members with dementia and “it is emotionally draining to take care” (Q33).

“More than anything, support. Not materially, but then emotionally because it is emotionally draining to take care” (Q33 - 74 year old, female, unemployed)

#### Money, transport and food

Almost half of the caregivers identified monetary needs that they require to assist with their daily household needs and caregiving tasks. Most caregivers stated that they needed money to “buy whatever [their family member] needs” (Q34) because the grant received from the government is insufficient and there is “no money to buy food” (Q35-Q37). Furthermore, a few caregivers required transport services for easier access to healthcare facilities or a car to “get around” as independently as needed (Q38-Q39) and a minimal number of caregivers mentioned needing employment so that they could have the financial means to hire a caregiver for assistance (Q40) and to alleviate their financial struggles (Q41).

“…for me I need, what is needed is transport and money. You know, the money for grant is too little” (Q35 - 51 year old, male, unemployed)

“Because she can’t go maybe in a long queue maybe from here to there, the feet is painful, it’s sore. So we need the transport to take her inside the clinic” (Q38 - 22 year old, male, unemployed)

“What is more for me, I wish I was working so that I could have a caregiver” (Q40 – 41 year old, female, unemployed)

“As a caregiver as I am not working, to be honest, I really need a job because financially I am struggling. I am really struggling financially” (Q41 - 50 year old, female, unemployed)

### Theme 3: Mobilizing the community

#### Awareness, knowledge and education

Most caregivers were of the opinion that a sense of awareness was needed within the larger community through information dissemination and education (Q42). Caregivers specified that education on the signs and symptoms of dementia would facilitate earlier detection, diagnosis (Q43) and a level of understanding that would assist others to approach families of people living with dementia with sensitivity (Q44). Furthermore, “awareness and more knowledge” would also serve to mitigate against cultural/spiritual perceptions of dementia and perceptions of exploitation or accusations of neglect if the community “understand [s] the sickness” (Q45-Q46). In addition, caregivers thought that the community needed practical “strategies of how to handle their people with dementia” (Q47) via the workshops conducted by *Alzheimer’s SA*. This would lead to community mobilization to promote awareness, knowledge and education (Q48).

“So I think they need better education on Alzheimer’s, the signs, because when it starts off, you don’t see it, you just think that this person is being silly or, you know, you just mean but yes, so just education on seeing the starting signs of it. That hey, maybe you know something you need to check up on it. And where too because I don’t think people know where they can go to, to get diagnosed” (Q43 - 40 year old, male, employed)

“They should get, they should be knowledgeable about this and be taught and be given strategies of how to handle their people of Dementia, Alzheimer’s because they, they do not know. They do not. They are ignorant and we cannot blame them. So, they need proper education” (Q47 - 49 year old, female, unemployed)

“First on the list we must all go for that two day training, that workshop, whatever they call it… [as] caregivers we come together we go through the training. Once we’ve got through that training then we can start getting other people, we go to the clinic…” (Q48 - 60 year old, female, unemployed)

#### Community initiatives

Half the caregivers in the sample went on to suggest various possible community initiatives to consider as provision for future dementia services are made. Suggested initiatives included a transport service for those who do not have vehicles or closer proximity of services for ease and accessible utilization for those struggling financially (Q49-50). Furthermore, caregivers suggested “bringing people together” to attend workshops to create awareness and to identify other families who are struggling with the manifestations of dementia in their homes – unbeknown to them – which may result in abuse of the older family member. This is vital and hinders help-seeking or treatment due to being “misunderstood and neglected” (Q51-Q52). As such, some caregivers proposed collaboration with “community radio stations”, “community newspapers”, and local clinics to meet their objectives (Q53). Additionally, other caregivers suggested health literacy campaigns that focus on dementia as other chronic illnesses, such as “HIV, TB and STIs” are (Q54-Q55). Similarly, a “helpline for dementia” was suggested for easy and immediate access to guidance and support for caregivers in the event of a “situation” (Q56). There were also suggestions for daycare facilities and activities for people with dementia to keep them stimulated, active and happy (Q57-Q58).

A few caregivers mentioned the importance of collaborating with government departments to recognize and prioritize funding to focus on dementia as a public health concern (Q59-Q62), thereby meeting the needs of families and their family members with dementia and creating services conducive thereof.

“The recommendation of dealing with this, it would be based on the services, we need closer services. The services must be close to us, and then be accessible to everyone” (Q50 - 52 year old, female, unemployed)

“Then we run with that but I want do something in September, yes it is late but at least if we can have a few tasks and community radio stations and maybe have a write up in one of the community newspapers I don’t know, I really don’t know that maybe dedicate two three days, you know every week, one day a week go to the clinic” (Q53 - 66 year old, female, unemployed)

“The same way or the methods to make people aware of HIV and TB and STIs and condoms and what-not, I think there should be the same amount of efforts and campaigns for dementia because it’s also something that people deal with. And the worse thing is unlike other illnesses, I’m not saying other illnesses are better, but then when you have TB or when you have HIV, the only time people have to take care of you is when it’s critical” (Q55 - 39 year old, female, employed)

“I think we need more support from the government also. Because like as we uplift people is like this thing is not being recognized. And the system is failing us, it is in most cases” (Q61 - 64 year old, female, employed)

“No I just wish that the government would take Alzheimer’s as seriously as they do HIV and those other diseases because just because they old doesn’t mean that, and it’s not just old people now that are getting dementia. It’s the younger people also so they really need to take it seriously” (Q62 - 52 year old, female, unemployed)

### Theme 4: From caregiver to caregiver

#### Get educated

When participants were asked if they had an important message for new caregivers of family members recently diagnosed, seventeen caregivers emphasized getting as much information as possible (Q63). Caregivers conveyed that education is key to generate “full knowledge” in order to understand and accept “who [they are] dealing with and what to do” (Q64-Q65).

“I will recommend that they will have to be educated about this and we have knowledge, full knowledge about it, so that we know who we’re dealing with and what to do” (Q64 - 40 year old, female, unemployed)

#### Reach out and use services

Twelve participants recommended that new caregivers reach out, find and access services available such as the support group at *Alzheimer’s SA* (Q66). They encouraged doing “research” if there is a lack of understanding and accepting help from others that can assist (Q67).

“To go out and seek for help and research. If you see something that you don’t understand, go and make a research. And if you find there is people that help you, go and look for that help. It will help you” (Q67 - 74 year old, male, unemployed)

#### Preserving dignity

Fifteen participants reflected on the qualities that caregivers needed to embody to preserve the dignity of their family members with dementia. Specifically, caregivers were emphatic in expressing the patience, understanding, love, compassion that is needed to take care of someone with dementia especially when challenging behaviours arise. In turn, caregivers should remain impervious to any “mean” things that their family member with dementia might say and “learn ways to keep [themselves] calm” (Q68-Q70). Furthermore, a caregiver expressed the “value” that older people possess as “a living library” and that they should not be seen as “this old frail person” – a sentiment that the youth should appreciate (Q71).

“To new caregivers, I think they…should be patient, they should be understanding, they should be more kind towards that person, more loving you see that’s the only way to deal with that person, if make sure they knew that you see, have an open heart, have an open heart” (Q68 - 52 year old, female, unemployed)

“They can be really, really aggressive and mean. So I don’t know. Maybe it’s, I guess it’ll be easier for them to separate themselves that whatever she’s saying or whatever the patient’s saying is not directly aimed at me. Just that it’s the disease talking. It’s not that person. Not evil, yes just have compassion and understanding and listen and learn ways to keep yourself calm” (Q70 - 58 year old, female, unemployed)

“The youth. I think that the elderly still have value. I mean, I think that’s something that young people don’t realise that the elderly have value, they are a library, a living library. So, they shouldn’t look at this, ag, this old frail person. They can teach you a lot about yourself, about the world. Ja, there’s a lot you can learn from them” (Q71 - 72 year old, female, unemployed)

## Discussion

This paper forms part of a larger study, in exploring the lived experiences of family dementia caregivers in a South African township. The specific objective of this component of the study was to give family caregivers an independent platform on which to reflect and identify their needs in the role of dementia caregiver. Our findings present a brief assessment of community resources, followed by individual needs that caregivers identified. Thereafter, caregiver ideas for mobilizing their community and advice to other caregivers are introduced.

As reported among other South African low-income samples (Hendriks-Lalla & Pretorius, 2018; Mahomed & Pretorius, 2020), almost every family caregiver in this study accessed their nearest primary or tertiary healthcare facility as their first point of contact after noticing a change in their family members’ behaviours. Even though these services were easily available, caregivers described oscillating between health facilities until they received a diagnosis. The challenges associated with service facility use are beyond the scope of this paper, and are discussed in Mahomed and Pretorius (2022). After diagnosis, most family caregivers reported being unaware of dementia-related support services and received no further information to access any services. This was synonymous with another South African study among male caregivers in a low-income community that stated “…during early stages of the disease, these caregivers reported being completely unaware of the services that were available to them and expressed their frustration at the lack of directives…” (Mahomed & Pretorius, 2020, p. 640), and international literature that also acknowledged the lack of information in hindering access to services (Dongxia Xiao et al., 2014; Singh et al., 2014; Stephan et al., 2018). This highlights the need to educate and orientate caregivers at the time of diagnosis to available service options (Kumar et al., 2019; Roberts & Struckmeyer, 2018).

Moreover, even though some caregivers had no knowledge of support services, they described their efforts in discovering them. As with the caregivers in Mahomed and Pretorius (2020), day care and respite services were found through family, friends and their own searches. The caregivers in this study only found *Alzheimer’s SA* – through informal referrals by others in the community, information obtained via television programmes or through their own internet searches. Of significance, there were no discoveries of respite or day care facilities in Soweto, like those found in low-income areas in the Western Cape (Mahomed & Pretorius (2020). This highlights the unequal distribution of resources in different geographical locations in South Africa – albeit enduring similar socioeconomic conditions.

Nevertheless, even though resources such as *Alzheimer’s SA* are deemed underutilized in communities (Jennings et al., 2015), this was not the case among caregivers in this study. *Alzheimer’s SA* was perceived by caregivers as a valuable service, where most family caregivers specifically appreciated the psychoeducational interventions at clinics, through workshops or via home visits once they became aware of these services. Of note, this sample had reported receiving information about dementia at their primary health clinic or healthcare facility through *Alzheimer’s SA*, while Hendriks-Lalla and Pretorius (2018) highlighted a lack of dementia-related information at primary health care clinics among their sample altogether. Furthermore, almost a third of this sample utilized the support groups that *Alzheimer’s SA* offered, which appeared to have empowered these caregivers to feel connected to each other, express and manage their difficult emotions. The use and value of support groups as perceived by South African family caregivers were also highlighted in Mahomed and Pretorius (2020). In contrast, there was a minority of caregivers in this study who did not use any services, such as the support groups at *Alzheimer’s SA.* Apart from having no knowledge of this service, these caregivers did not think that this type of support met their needs (Ducharme et al., 2011; Stephan et al., 2018) and did not find it useful because they seemed to experience it as repetitive and/or monotonous by implication.

Regarding the individual needs that caregivers identified, half the sample indicated the need for respite care to reduce caregiver burden, facilitate well-being and to allow for caregiver autonomy for leisure activities when needed. The need for respite services for these outcomes have been well documented in informal caregiving literature (Han et al., 2020; Hendriks-Lalla & Pretorius, 2018; Kumar et al., 2019; Mahomed & Pretorius, 2020; Park et al., 2015; Roberts & Struckmeyer, 2018). Furthermore, a prominent need that majority of the caregivers emphasized was all-inclusive psychoeducation and skills training. Caregivers made explicit their need for comprehensive education on dementia and how it presents as behavioural challenges amongst their family members who receive a diagnosis thereof. In addition, they verbalized that this knowledge, accompanied by practical training, would equip them with the skills needed to manage and respond appropriately to their family member’s as their needs change through the disease process (Gitlin & Hodgson, 2016; Mahomed & Pretorius, 2020; Ramirez et al., 2021; Sadak et al., 2017), and at their disposal during a crisis situation (Jennings et al., 2015). Interestingly, caregivers also expressed the need for more information so that they are better able to acclimatize and reflect on what their needs are. By implication, without in-depth, easy- to- access information it seems caregivers do not know what they *should* need until they know the extent of what they are dealing with (Roberts & Struckmeyer, 2018) – fostering a sense of inadequacy and low confidence in their capacity to manage their caregiving role (Hendriks-Lalla & Pretorius, 2018; Jennings et al., 2015; Ramirez et al., 2021).

The need for emotional support due to the demands of dementia caregiving has been well established in the literature (Gurayah, 2015; Han et al., 2020; Hendriks-Lalla & Pretorius, 2018; Wang et al., 2014). Of note, although the presence of caregiver grief and psychological distress was reported on in the larger study (Mahomed & Pretorius, 2022), caregivers did not identify emotional support among their prominent needs.

Of significance, almost half the sample of caregivers in this study expressed their needs for financial assistance, transportation and food. These needs were also made explicit in a South African study among caregivers in a rural area (Gurayah, 2015). Specifically, caregivers clarified that the stipend that they relied on from the government was insufficient to meet their basic need for food as well as their family member’s additional needs such as transport to access healthcare or support services. Once again, even though majority of the caregivers in this study were unemployed (Mahomed & Pretorius, 2022), the need for employment opportunities was not eminent.

Compelling findings of the study were caregivers’ ideas on mobilizing their community through awareness, knowledge and education and suggested initiatives. Majority of the sample in this study believed that information dissemination and education was vital to create awareness of dementia within the larger community of Soweto, consistent with other South African and international samples regardless of socioeconomic conditions (Griffiths & Bunrayong, 2016; Han et al., 2020; Mahomed & Pretorius, 2020; Mkhonto & Hanssen, 2018). This was important to caregivers as this would result in earlier detection and diagnosis (Kumar et al., 2019; Mahomed & Pretorius, 2020; Ramirez et al., 2021; Roberts & Struckmeyer, 2018), which would assist in fostering understanding and sensitivity towards families with relatives of dementia. Additionally, if the community is aware of and educated about dementia, this would serve to “eradicate the witchcraft beliefs connected to severe dementia” (Mkhonto & Hanssen, 2018, p. 169) and relieve caregiver fears and insecurities around accusations of exploitation or neglect of their family member (Mahomed & Pretorius, 2020). Additionally, caregivers also identified the need for practical strategies (Park et al., 2015) via workshops conducted by *Alzheimer’s SA* that should be taught on a larger scale in the community. According to caregivers, workshops would also facilitate awareness and help identify other families in the community who are struggling with the behavioural changes of their family members and prevent abusive behaviours towards them, which will likely impede their access to support and services (Cipriani & Borin, 2014; Mukadam & Livingston, 2012). Moreover, caregivers suggested a transport service for those unable to afford travel costs to service facilities or do not have access to vehicles.

Furthermore, prospective collaborations with media outlets such as community radio stations and newspapers, initiatives such as health literacy campaigns (Wang et al., 2014), a helpline for dementia, and service provision for day care facilities offering both respite and activities for family members with dementia was suggested to build community resources and infrastructure (Gilbert et al., 2017; Mahomed & Pretorius, 2020; Roland & Chappell, 2015). Importantly, a few caregivers highlighted the need for government intervention in this regard, to assist with funding and prioritize the needs of families affected by dementia as a national public health concern (Hendriks-Lalla & Pretorius, 2018; Mahomed & Pretorius, 2020; Wang et al., 2014).

Finally, caregivers offered advice that they considered most pertinent to assist new family members in their role of dementia caregiver. Most caregivers primarily encouraged new caregivers to seek out information regarding dementia to fully understand the magnitude of the journey ahead. Furthermore, some caregivers recommended reaching out for help to find and access support services and being open to accepting assistance from others. The last piece of advice that half the caregivers in this sample offered, focused on the quality of care and preserving the dignity of people living with dementia (Roberts & Struckmeyer, 2018). Caregivers considered qualities such as love (Hendriks-Lalla & Pretorius, 2018), understanding, patience and compassion (Mahomed & Pretorius, 2020) as core values to embody especially when confronted with challenging behaviours. Furthermore, the notion that older people should still be revered despite their deterioration is an invaluable sentiment worth advocating for.

## Conclusion and recommendations

This study specifically aimed to ascertain the needs of dementia family caregivers in a township through their own lens – without inferring needs through professional assessments of caregiver needs as usually reported in caregiving literature (McCabe et al., 2016). This is an important step to ensure that dementia service provision in townships and low-income communities are aligned with the support and service needs that caregivers themselves identify. The need for information permeated across all themes and subthemes, demonstrating its importance on multiple levels. Our findings demonstrate that Black African caregivers in townships need: sufficient information and orientation to dementia-specific services on diagnosis, psychoeducation on dementia as a disease, its behavioral manifestations and practical skills to manage the disease process with their family members in light of their unpredictable behaviours and any emergency situations that may occur. Furthermore, in-depth, easy-to-access education is needed to equip caregivers with the confidence and fortitude that they require to cope with the magnitude of their task as dementia caregiver. The need for emotional support and employment – which would likely be identified and deemed important by researchers and professionals – was not identified as such by caregivers themselves, thus demonstrating the ease at which incompatible, professionally-inferred versus subjective needs contribute to the unmet needs of dementia caregivers in communities.

Additionally, family caregivers proposed ideas to mobilize their community through initiatives that would foster awareness, knowledge and education to facilitate earlier detection and diagnosis of dementia. According to caregivers, this would serve to cultivate empathic understandings towards families with relatives of dementia, mitigate spiritual/traditional beliefs associated with dementia, and relieve caregiver fears and insecurities associated with accusations of exploitation or neglect of their family members. Moreover, caregivers emphasized the need for informational and educational workshops for the larger community to assist with identifying families who may be suffering in silence and to facilitate their access to support services. A transport service, helpline for dementia, day care facilities, media campaigns and collaborations with government to obtain funding and initiate policy change was suggested to build community resources and infrastructure. Lastly, from caregiver-to-caregiver, their proposed advice explicitly encouraged new caregivers to seek out comprehensive education, support and services and to preserve the dignity of their family members with dementia – who should be revered and treated with compassion, love, understanding and patience.

Our caregivers’ needs and ideas for community initiatives align in striking similarity to most priority areas that ADI identify as components of any National Dementia Plan that should be included among a country’s policy and legislation – which South Africa does not have. These priority areas include: the promotion of awareness and education, early detection and treatment, provision of home-based support services and residential care, better referral pathways and training initiatives for professionals, and research and technology innovation to facilitate national transformation of dementia care (Alzheimer’s Disease International, 2018). Thus, efforts to create a National Dementia Plan for South Africa should take precedence in collaboration with various stakeholders in communities, policy-makers, multidisciplinary teams of health professionals and most importantly – giving a voice to individuals and families directly affected by dementia.

Realistically, since the creation of a National Dementia Plan for South Africa could take years, it is recommended that efforts to mobilize and support existing community resources – albeit limited – should commence. This includes resources such as *Alzheimer’s SA,* family caregivers themselves and health professionals in public service who are willing to actively participate in advocacy and training initiatives as proposed. Efforts in this regard are being made by the first author of this paper, who is in the process of establishing a memory clinic with her team to service low-income areas East of Johannesburg, South Africa.

## Limitations

Findings have not drawn inferences about correlations between caregiver demographics such as relationship status, age, gender or culture and perceived needs specific to these variables. Perhaps this is an additional lens through which future studies may approach this area as a research question. Furthermore, specificity in terms of the stage of disease progression has not been linked to caregiver needs. This is perhaps due to insufficient information and ambiguous understandings reported by caregivers in this paper, as well as in Mahomed and Pretorius (2022), which made it difficult for caregivers to demarcate. Future studies should aim to address these limitations after psychoeducational programmes are implemented and caregivers are better able to identify their needs through different stages of the disease process. Furthermore, in order for a National Dementia Plan to be initiated, future studies should be conducted on a larger scale among various subsets of the South African population across multiple communities.

## Supplemental Material

Supplemental Material - Giving voice to the voiceless: Understanding the perceived needs of dementia family carers in Soweto, a South African township Supplemental Material for Giving voice to the voiceless: Understanding the perceived needs of dementia family carers in Soweto, a South African township by Aqeela Mahomed and Chrisma Pretorius in Dementia.
