# Supplementary material for: Giving voice to the voiceless: Understanding the perceived needs of dementia family carers in Soweto, a South African township
Source: Dementia (London). 2024 Feb 14;23(4):622–42. doi: 10.1177/14713012241234155 (PMC11059838; doi:10.1177/14713012241234155)
Supplement: Supplemental Material - Giving voice to the voiceless: Understanding the perceived needs of dementia family carers in Soweto, a South African township [file sj-pdf-1-dem-10.1177_14713012241234155.pdf]

Supplemental Material: Illustrative Quotes

Broad Theme	Subtheme	Illustrative Quotes
**Theme 1: Assessing Community Resources**	*Available Services*	"Services, I take her to the clinic" (Q1)<br>"Okay. That's when we say "okay, she's not okay" and then we went to the clinic but still it took time because you know clinics, they didn't like transfer us to the doctor that she is in right now. It took us surely a year doing the same thing over and over again" (Q2)<br>"Because first, we didn't know. First, I remember I used to take her to Bara, and then from Bara, they didn't help us, and then back to the clinic. And then we went to the clinic, went to the clinic, until one doctor referred us to the mental [health] clinic" (Q3)<br>"Like we went to a doctor, there's a doctor she went to who recommended a psychologist, and when we got that, we then heard of this a week later. My cousin, she told us about this and then she came in with her mother and they came and reported this. So we haven't really had any services – this is our first sort of contact…" (Q4)
	Services after diagnosis	"No services at all for what I can say that I knew of" (Q5)<br>"No there were no services, the only thing is they brought her some tablets she drank them but they had no effect you see" (Q6)<br>"There isn't much information out there - there is nowhere to go" (Q7)<br>"I found out on the internet. Some other people talking about it" (Q8)<br>"None. Nothing. Nothing at all. Everything I found I had to look for, I had to really look for it (Q9)<br>"Where I saw a program on TV and I think that's everything there it was almost similar to what I was experiencing at home, and I got the name there of the people that were responsible and I called them" (Q10)
	Helpful Services	"It was teaching us about the dementia, how to take care of the person who has dementia and how the difference between a normal, what do you call, old person and a dementia person. Yes. in the normal for, if you forget, it, sometimes its normal and then there's a difference between a person who forgets with dementia. That person will forget and not knowing it all away where is something. It is teaching about how to take care of elderly people . How to wash them and how to give them food, water, maybe sometimes you should use colouring things. So that the person can enjoy the water. It's so, it was so good" (Q11)<br>"For them to make me to accept the illness and how I should behave towards the person. What to do. The tips when I want to you know wanting him to do like bathing. What should I do if sometimes it was not easy? You know that kind of thing and the love that should give to the person" (Q12)<br>"Non-medical services, I think the counselling, being able to talk to someone who understands like what's going on. I like that they come to you to the patient house. They talk to the family. I

		actually invited her sister and her husband to come and also listen try to a better understanding of what, what it is and how to communicate with that person from now, just to make things better. And the support groups help a lot also" (Q13)<br>"I think for her being around and checking on us and coming and speaking to us as a family – it has been helpful because I think if I didn't find her I wouldn't have known even today that there's damage here and Mom is sick of dementia and whatever – I wouldn't have known" (Q14)<br>"Yes, I like to come here because my mind is concentrated and I feel happy when I'm here. We share everything. If I've got a problem I tell them, we are sharing to solve the problem" (Q15)<br>"Yes because when we come her in support group we will talk about those thing, so they help me not to be angry, angry over you know" (Q16)<br>"Yeah, before yeah I don't want to lie. Before the Clinic it was helpful with their pills, because she was missing, going up and down, they give me pills" (Q17)<br>"Those, those tablets or medication that can make her be more manageable" (Q18)
	No Service Use	"I did not know about the support groups" (Q19)<br>"Yes, she told us that there is support groups and whatever but we have never been in one of them" (Q20)<br>"No, I didn't ask for help though" (Q21)<br>"Well, I find that I don't need to because it's the same people and it's the same stories" (Q22)
**Theme 2: Identifying Caregiver Needs**	*Respite Care*	"…sometimes I need to have my own space. You know so that I can rejuvenate myself, you know because sometimes it is exhausting to take care of that person and even weekends you are still there, we have to go to church together and come back. Monday, Tuesday, Wednesday, Thursday. You know you are doing the same thing every day. So you don't get rest…" (Q23)<br>"So people who are caregivers need help… so sometimes they need to go out and see other people and to socialize, unlike being there facing your problem 24 hours and not getting a break from it" (Q24)<br>"Maybe just somebody to help me, to help me ja, just to say okay I just need a break, let me just go to such and such a place ja" (Q25)
	Psychoeducation and Skills Training	"And yeah and someone like to come and teach us more about dementia, the symptoms because some people like my Mom … we didn't know that she had dementia. We just know that maybe she was joking or you know those kind of things" (Q26)<br>"Training as well, training and ja knowledge, knowledge to know what you're dealing with, so that you know…mom is doing this things like nothing's wrong with her, she performs but who wants to cry and do funny things and remove things from the kitchen and put them in the bedroom hey? Seems like it lacks knowledge" (Q27)<br>"I think as a caregiver you need to be aware, because I was a caregiver not aware of what she was going through at the time, but once I became aware that made I easy for me to live with a person and to know how must I treat her" (Q28)

		"I think it's education, education for the caregivers, because when you ask me what do I need to help take care of mum, maybe with the right education or enough knowledge of the illness and what is being used with other patients at home, then I would know that, okay, we don't have this, we need this, we don't have this, we need that" (Q29)<br>"…they don't give you advice on what you should do, how to handle the person, what not to do you see, they don't give you that on how to take care of [a] situation when it happens…" (Q30)<br>"A lot of information, and if I have questions, being able to access help quick, resources that will make our lives easier" (Q31)
	Emotional Support	"As a caregiver we need a counselling because sometimes you cannot cope, I am not going to say as I know that my mother is with dementia. There are other challenges you know that you cannot cope. You know and then sometimes you will need someone to talk to you know"(Q32)<br>"More than anything, support. Not materially, but then emotionally because it is emotionally draining to take care" (Q33)
	Money, food and transport	"Money would make my life easy (laughing), because obviously you are able to buy whatever she needs, you are able to take care of things better" (Q34)<br>"…for me I need, what is needed is transport and money. You know, the money for grant is too little" (Q35)<br>"Like money, you know, there's never enough money. Ja, basically it's the money" (Q36)<br>"Financially as I said you cannot go cry outside your people about your financial situation. Sometimes there is no money to buy food" (Q37)<br>"Because she can't go maybe in a long queue maybe from here to there, the feet is painful, it's sore. So we need the transport to take her inside the clinic" (Q38)<br>"Like a simple one is a car – a simple on. Just going around and getting around, that's I simple one that I need" (Q39)<br>"What is more for me, I wish I was working so that I could have a caregiver" (Q40)<br>"As a caregiver as I am not working, to be honest, I really need a job because financially I am struggling. I am really struggling financially" (Q41)
**Theme 3: Mobilizing the Community**	*Awareness, Knowledge and Education*	"A lot of information is needed with the community" (Q42)<br>"So I think they need better education on Alzheimer's, the signs, because when it starts off, you don't see it, you just think that this person is being silly or, you know, you just mean but yes, so just education on seeing the starting signs of it. That hey, maybe you know something you need to check up on it. And where too because I don't think people know where they can go to, to get diagnosed" (Q43)<br>"This has it in the community maybe like you need to teach people the symptoms what is, so that

		they know. When they come they will be like "hi she will be fine" and those things and that kills my Dad" (Q44)<br>“Awareness and more knowledge so that we’re able to deal especially…maybe I’m accusing my siblings for not taking care of mum cause they’re scared but I don’t I’m not too sure if they scared, I didn’t have information as much as I may I didn’t know much about Dementia but I had to educate myself and mix with other people who have same condition and with the black community, if we have this, sometimes they’ll say you mad or you’re a witch or whatever, but we have to dig deeper and search deeper, to deal to have to assist your parent” (Q45)<br>“I think the understanding of the sickness. Yes that was like what the most the community needs. Because since I understand the sickness, now I can see most of the elderly there have got the sickness. I think the community needs to be communicated about this because otherwise they say we are mad, others they say no we doing it purposefully” (Q46)<br>“They should get, they should be knowledgeable about this and be taught and be given strategies of how to handle their people of Dementia, Alzheimer’s because they, they do not know. They do not. They are ignorant and we cannot blame them. So, they need proper education” (Q47)<br>First on the list we must all go for that two day training, that workshop, whatever they call it…[as] caregivers we come together we go through the training. Once we’ve got through that training then we can start getting other people, we go to the clinic…” (Q48)
	Community Initiatives	“And then I think sometimes transport, it might be a problem to the community, to our community, because people, some other people they don't have cars and the money for the grant is too little, they cannot afford to sustain themselves all through the month and put other money for transportation. I think transport is essential. Or the services must be closer to the people” (Q49)<br>“The recommendation of dealing with this, it would be based on the services, we need closer services. The services must be close to us, and then be accessible to everyone” (Q50)<br>“You know we need to bring people together under one roof and start having these workshops…we tell especially the young kids because we have kids that are taking care of the old ladies old men who are their grandparents, you know what happens, you have these youngsters that dump their kids with their parents and it’s sad to finish but these kids don’t understand when they don’t get proper meals, they don’t have cooked food, they don’t understand what’s going on at home because this old woman has now suddenly got [dementia], so we need to sensitize people to find out how many homes have people that are suffering, how many people are taken care of and some people have people who are sufferers but they don’t understand what it is that needs to be done, some get abused I’ve got to learn that there will come a time when my mom will not want to take a bath, so then it triggered something, it made me realize that okay now that I see people, old people that have not had a bath, they could be going through that. So the minute we start, we go to the radio stations, community radio stations, they call them to say we have these meetings, come let’s talk, let’s share. I tried also to get people

		who've gone through this journey who have had experience to come share with us their experiences and how can we work together you know just to spread the word and tell other people" (Q51)<br>"I think a lot of workshops must be done in the communities, and people must be made aware of the dementia and how to recognise it. Because I think other people – because of lack of knowledge – they have family members with this challenge but they are not getting proper help from the systems or support because I think they are misunderstood and just neglected. They are not given the attention that they deserve" (Q52)<br>"Then we run with that but I want do something in September, yes it is late but at least if we can have a few tasks and community radio stations and maybe have a write up in one of the community newspapers I don't know, I really don't know that maybe dedicate two three days, you know every week, one day a week go to the clinic" (Q53)<br>"..to be very honest… I think we need health literacy. I'm going to be very honest. We need to be coming together and mobilizing around health knowledge, knowing what's healthy, what's unhealthy" (Q54)<br>"The same way or the methods to make people aware of HIV and TB and STIs and condoms and what-not, I think there should be the same amount of efforts and campaigns for dementia because it's also something that people deal with. And the worse thing is unlike other illnesses, I'm not saying other illnesses are better, but then when you have TB or when you have HIV, the only time people have to take care of you is when it's critical" (Q55)<br>"…just human support. Maybe a, I don't know if there is a helpline for dementia or what support for people who are taking care of people for dementia. You know, you can just call just like when maybe you are raped or abused – there are help lines. Something like a call centre where someone is always standing by to take your call and say what is happening, what's going on, okay I think you need to do 1, 2, 3 – maybe calm me down when maybe when I'm panicking about a situation or whatever the case may be" (Q56)<br>"Or maybe we have a place in the community where we take them during the day. Because I think, also, because what I've seen, she would sit, she would like to sit in her corner, she will get into a corner and sit there. It doesn't matter – she can sit there for the whole day. And she's not talking to anyone – she's talking to herself and all those things. If we would have in our community places whereby we will take them during the day, maybe they will do something. Speak and maybe teach them some things or do some exercise that, like in the afternoon take them back home, I think that would help as well" (Q57)<br>" …if there could be activities, social gatherings for old people, activities, I think it would make a difference because even their dementia would lessen because of why? They can meet with the people similar to the kind of situation they are going through and they could share ideas and realize that it is not only her and talk about that…I think it would make it, it would make life better for people that are taking care of people with dementia because of at least they would have activities outside of their home environment where they can be happy, laugh and talk about

		things and have whatever, a dance for them and all that and then when she comes home maybe she can talk about what was happening and all that. I think that would make a difference" (Q58)<br>"I just wish that places like Alzheimer's South Africa get funding so we can educate a whole more people. Like it would be nice if they had like a space in a clinic, or, you know. So, like a social worker has an office there, they could have an office there to help people" (Q59)<br>"Social development department – maybe. They must bring us together, we must talk amongst ourselves and there must be a control and awareness of such people with their needs. And then maybe the, our pleas and requests, pleas, should be taken to Social Development – maybe we could get some help" (Q60)<br>"I think we need more support from the government also. Because like as we uplift people is like this thing is not being recognized. And the system is failing us, it is in most cases" (Q61)<br>"No I just wish that the government would take Alzheimer's as seriously as they do HIV and those other diseases because just because they old doesn't mean that, and it's not just old people now that are getting dementia. It's the younger people also so they really need to take it seriously" (62)
**Theme 4: From Caregiver to Caregiver**	*Get Educated*	"Like they should try and get as much as they can, get information as much as they can" (Q63)<br>"I will recommend that they will have to be educated about this and we have knowledge, full knowledge about it, so that we know who we're dealing with and what to do" (Q64)<br>"I think they should take it easy and then accept the situation nè and understand what is going on. It is more important and read more about dementia so that they may understand and then yes, they should take care because there is nothing we can do and there is nothing, when dementia is there, we cannot turn it back" (Q65)
	Reach Out and Use Services	"I would recommend them to come to our support group. They must use this facility and this kind of facility should be all over Soweto" (Q66)<br>"To go out and seek for help and research. If you see something that you don't understand, go and make a research. And if you find there is people that help you, go and look for that help. It will help you" (Q67)
	Preserving Dignity	"To new caregivers, I think they…should be patient, they should be understanding, they should be more kind towards that person, more loving you see that's the only way to deal with that person, if make sure they knew that you see, have an open heart, have an open heart" (Q68)<br>"They need to really be understanding and be accepting of what is happening and supportive of the person, and not judge them or be impatient with time they … because they can be repetitive, repetitive questions or whatever. Patience is needed, a lot of patience is needed to take care of someone with dementia" (Q69)

		“Patience, learn patience and compassion and love what you do will love, love the person that you're looking after. Because I think you can’t, you know it is not like looking after a dog or something, you have to really care about the person in your heart of hearts to look after them properly. I don't know how people who are hired look after a stranger. It's weird to me like how do you care for a stranger? And because they are going to really dig deep and be really mean. They can be really, really aggressive and mean. So I don't know. Maybe it's, I guess it'll be easier for them to separate themselves that whatever she's saying or whatever the patient's saying is not directly aimed at me. Just that it's the disease talking. It's not that person. Not evil, yes just have compassion and understanding and listen and learn ways to keep yourself calm” (Q70)<br>“The youth. I think that the elderly still have value. I mean, I think that’s something that young people don’t realise that the elderly have value, they are a library, a living library. So, they shouldn’t look at this, ag, this old frail person. They can teach you a lot about yourself, about the world. Ja, there’s a lot you can learn from them” (Q71)
